# Annotations

**Published:** 1901-01-26

**Authors:** 


### ANNOTATIONS.

### Plague in Hull.

THE apparent ease with which an outbreak of plague was made to fizzle out in Glasgow has given the country confidence, so that the announcement made last week of the occurrence of several cases of this disease on a vessel at Hull has been received with considerable equanimity. All the same the attack was one of very great virulence, all the patients on the ship quickly dying, and the disease being in its most infectious form, that of pneumonic plague; and had the time of year been different, or had the sanitary officials been less vigilant, the affair might have turned out much more seriously than, so far, it has done. Hull, like Glasgow, has the good fortune to possess an able and energetic medical officer of health. The citizens of Hull will not readily, we hope, forget what Dr. Mason did for them a few years ago when cholera got a footing in their midst, for their experiences of cholera in previous epidemics had been terrible; and we have no doubt that if plague should threaten they will again, as in those days, give him a free hand. In the meantime it must be noted that, as has so often happened before, in this instance also the disease was not diagnosed as plague until the number and fatality of the cases excited suspicions. If a smaller number had been affected, or if death had not taken place, the first diagnosis, namely, that of influenza with lung complications, would certainly have been accepted, and there would have been but little to prevent the infection being carried on shore and becom-ing diffused before the nature of the malady was discovered. This happened at Glasgow, and this has been the course of events in almost every case in which plague has been intro-duced into a new country. We cannot then trust to any machinery which aims at keeping plague out of the country by inspection. When this ship arrived in Hull, although there was a dead man on board, there was nothing to show that he had died of plague, and there was apparently nothing to have prevented the four men who died taking train to four distant inland towns before they showed signs of illness, and thus starting four separate foci of infection. Our only protection, then, is an ever-ready sanitary organisation throughout the country, prepared to cope at any time with any outbreak of any infectious disease.

